# A review on extraction, isolation, characterization of bioactive compounds obtained from agri-food waste and their potential for industrial application

**DOI:** 10.3389/fchem.2025.1669737

**Published:** 2025-11-20

**Authors:** Joel B. Njewa, Maurice Monjerezi, Lucia Kabanga, Felix Kumwenda, Jimmy Sumani

**Affiliations:** 1 Department of Chemistry and Chemical Engineering, Centre for Resilience Agri-Food Systems, School of Natural and Applied Sciences, University of Malawi, Zomba, Malawi; 2 Department of Basic Sciences, Lilongwe University of Agriculture and Natural Resources, Lilongwe, Malawi

**Keywords:** Agri-food waste, extraction, purification, characterization, bioactive compounds

## Abstract

The increased global food demand has resulted into extensive agricultural activities to offset the demand. The agri-activities generates large volumes of agri-food wastes (AFW) which creates disposal challenges and environmental pollution concerns. However, agri-wastes possess essential bioactive compounds with industrial applications. The primary focus of the study is to discuss techniques used in extraction, isolation, purification and characterisation of bioactive compounds found in AFW and their potential industrial applications. Traditional and emerging extraction processes; solid-liquid phase, liquid-liquid phase, distillation, crystallisation, thin layer chromatography and gel filtration chromatography are used for purification and isolation of bioactive compounds. FT-IR, NMR, UV-Vis and GC-MS analytical techniques are usually used in characterisation of bioactive compounds. AFW are reported to contain high levels of bioactive compounds with excellent antioxidants properties and biological activities that are ideal for cosmetics, pharmaceuticals and nutraceutical industries. However, the scalability of the use of bioactive compounds from AFW in various industries face challenges such as the use of large volumes of solvents and reagents in the extraction process that are a threat to human health and cause environmental pollution. The occurrence of phytochemical compounds with different properties and characteristics presents difficulty during extraction and purification processes. It is suggested that the use of pretreatment methods, innovative biological techniques and building closed-up systems that aim to repurpose the AFW into new products can promote their scalability and reduce environmental effects.

## 1 Introduction

The increase in generation of agri-food waste (AFW) has widely been associated with global rapid population growth. Recent studies indicate that the global population is expected to reach 9.7 billion by the year 2050 and the demand for food is estimated to increase by 50 percent (Pakseresht et al., 2022). As such, different parts of the world practices extensive agricultural activities to close the existing food demand gap against the rapid population growth. Consequently, extensive agricultural practices have resulted into huge generation of agri-wastes estimated in the range of between 1.3 and 1.6 billion tons every year (Benyahya et al., 2022). Agri-food waste comprises of residues and by-products produced during the agricultural production and food processing activities, respectively (Voss et al., 2024). AFW are produced in large quantities of by-products that are generally disposed of without utilisation (Tapia-Quirós et al., 2022). Several studies have reported that if the wastes are not properly managed they create serious disposal challenges by causing environmental degradation such as air, soil and water pollution and greenhouse gas emissions (Lackner and Besharati, 2025; Oluseun and Adebukola Adebiyi, 2021; Phiri et al., 2024). It is indicated that, 19–29 percent of global greenhouse gases emission are associated with AFW improper disposal (Chauhan et al., 2018).

**FIGURE 1:**
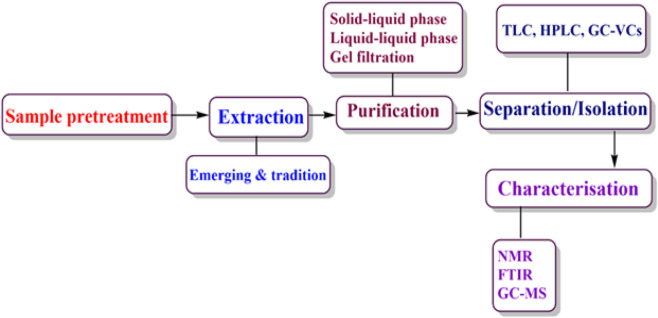
Extraction and characterization of bioactive compounds from AFW.

**FIGURE 2:**
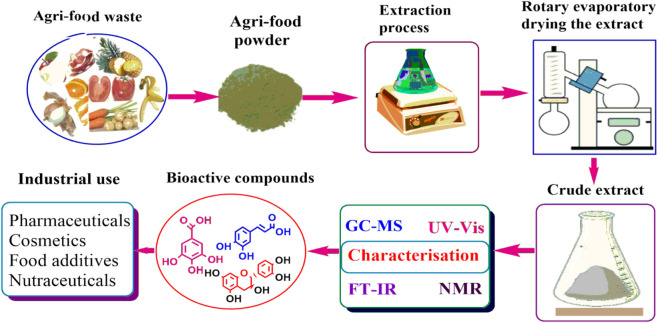
Pictorial representation of how bioactive compounds are obtained from AFW.

However, agri-wastes are also recognized as rich sources of bioactive compounds that have diverse applications in sectors such as pharmaceutical, cosmetic and food industries. Studies have shown that phytochemicals obtained from AFW possess high levels of bioactive compounds such as phenolic compounds, antioxidants and nutraceuticals among others (Castrica et al., 2019; Hrelia et al., 2023; Latella et al., 2024; Manousaki et al., 2016; Zhang and Zhang, 2024). The phytochemicals are associated with human health benefits such as antibacterial and anti-inflammatory properties that are responsible for alleviating the occurrence of chronic diseases such as cardiovascular diseases, cancer and diabetes (Rodríguez García and Raghavan, 2022; Sorrenti et al., 2023; Yadav et al., 2024). Additionally, the extraction of beneficial compounds from AFW helps to resolve environmental issues associated with disposal challenges whilst providing essential resources for manufacturing industries. As such, researchers’ interest and preference has risen towards searching bioactive compounds sourced from AFW. These compounds are obtained from AFW through sequential process as summarized in (Figures 1, 2). The processes include; extraction, purification, separation and isolation and characterization.

This review study therefore, aims to provide comprehensive extraction techniques, isolation and purification strategies and characterisation techniques for bioactive compounds obtained from AFW. Several techniques and protocols have been developed and explored to increase the yield of bioactive compounds obtained from AFW for various applications. The paper has discussed in greater depth the potential of bioactive compounds towards various industrial applications.

## 2 Methodology

The present study employed a literature-based examination that involved searching and downloading numerous published peer-reviewed papers related to the topic of study. The papers were accessed from various databases based on relevance to the study topic and publication date. The papers published recently were favoured and preferred. The papers were searched and downloaded from various databases (Web of Science, PubMed, Google Scholar, PLOS and ScienceDirect). The searching technique involved the use of keywords “Extraction techniques,” “Isolation and purification,” “Bioactive compounds,” “Agri-food waste” and “Characterisation,” among others.

## 3 Results and discussions

### 3.1 Extraction

Extraction process is fundamental towards obtaining bioactive compounds from various sources and classified into traditional and emerging. Traditional extraction techniques require the use of organic solvents, heat and continuous agitation. The maceration, soxhlet and hydro-distillation are recognized examples of traditional extraction protocols. On the other hand, emerging extraction techniques are recent analytical tools that aim to promote extraction efficiency and reduce the environmental effects associated with the use of extraction solvents and reagents (Hansen and Pedersen-Bjergaard, 2019). Microwave assisted extraction (MAE), ultrasound assisted extraction (UAE), enzyme assisted extraction (EAE), supercritical liquid extraction (SFE) and solid state fermentation (SSF) are common examples of advanced emerging extraction protocols (Alara et al., 2021; Mushtaq et al., 2014; Nn, 2015). Phytochemical compounds can be acquired from various AFW sources by employing extraction techniques. Studies have revealed that the most favorable and effective protocols of extracting and isolating bioactive compounds from AFW are supercritical fluid extraction, enzymatic assisted extraction, microwave assisted extraction, ultrasound assisted extraction and solvent extraction with green solvents. Recent studies have shown that the aforementioned methods have drastically promoted the recovery of bioactive compounds from AFW sources (Hu et al., 2023; Long et al., 2015; Parry and Hawkesford, 2010). These methods have led to the increase in the efficiency and yield to bioactive compounds (Fuertes et al., 2024). These methods are discussed in greater detail regarding their operating principles. However, the setbacks and advantages associated with each extraction protocol are summarized in (Table 1).

**TABLE 1:** Summarizes the bioactive compounds extraction protocols with their associated benefits and setbacks.

Methods	Advantages	Disadvantages	References
Supercritical fluid Extraction	High efficiency and selectivity for target compounds Environmentally friendly Suitable for thermal sensitive compounds	High operational costs Requires technical expertise	Kai Bin et al. (2020) Li et al. (2022)
Enzyme assisted Extraction	High yield and extraction efficiencies Favours specific compounds extraction Environmentally friendly	Enzymes are affected by pH and temperature Enzymes are expensive	Akyil et al. (2018) Yogeshri et al. (2023) Arya et al. (2019)
Microwave assisted Extraction	Shorter extraction time High yield and quality of extracts Requires less solvent	Degradation of sensitive compounds Equipment cost and complexity	Medina-Torres et al. (2017) Jovanović et al. (2017) Jovanovic et al. (2017)
Ultrasound assisted Extraction	Less extraction duration High yield and quality extracts Low use of solvent Less operation costs Ideal for heat sensitive compounds	Requires optimization High equipment cost	Cvetanović (2019) Medina-Torres et al. (2017) Saini et al. (2021)
Pressurized liquid Extraction	Less extraction duration Less energy consumption Less solvent use Automated	Optimization required Expensive Matrix effects Possible degradation	Yan et al. (2017) Chatzimitakos et al. (2023) Ranjha et al. (2021)
Solid-Phase Microextraction	Simplicity and sensitivity High extraction yields Compatibility	Limited sample capacity Fiber degradation Matrix effects	Bagheri et al. (2014) Mustafa and Turner (2011) Aulakh et al. (2005)
Pulse electric field Extraction	High efficiency High extraction yields Less energy consumption Less extraction duration	Degradation of compounds Expensive Requires optimization	Ranjha et al. (2021) Gavahian et al. (2018) Soriano et al. (2024)
Solvent Extraction	Cheap Less expertise Favours all solvents	Consumes more solvents Low yields Prolonged extraction period	Saini et al. (2021) Li et al. (2022) Arya et al. (2019)

#### 3.1.1 Solvent extraction

Solvent extraction is one of the traditional methods of extraction and relies on organic solvents which break down the plant matrix in order to extract bioactive compounds among other compounds. Solvents such as ethanol, acetone and methanol are commonly employed during the bioactive extraction process. Studies have shown that the quality of the bioactive compounds obtained and the efficiency of the extraction process depend on the solvent used in the extraction process. The study done by Soares et al. (2023) found that antioxidant levels and the chemical profile of extracts are influenced by the solvent used in the extraction process. The study recommended that suitable selection of solvent is necessary to maximize the extraction of bioactive chemical substances during the extraction process. Moreover, other factors such as temperature, extraction period, as well as, solid-to-liquid ratio are also recognized to have an effect on the solvent extraction efficacy that requires optimization in order to attain the highest extraction efficiency (Ben-Othman et al., 2020). Nevertheless, its wide use, the solvent extraction protocol is associated with some limitations, such as damage to heat-delicate compounds and environmental issues caused by the use of organic solvents. Recent studies have highlighted the benefits of using green extraction technologies that require lesser, non-toxic and environmentally friendly solvents (Panzella et al., 2020). Studies have demonstrated that green solvents are a promising alternative for obtaining bioactive compounds from plant-related materials without damaging the environment (Molina-Montero et al., 2023). Solvents such as ethanol and water are commonly used to obtain preferred bioactive compounds from AFW. Studies carried out on grape pomace peels through the use of ethanol and water mixture successfully extracted anthocyanins, trans-resveratrol and quercetin phenolic compounds (Brazinha et al., 2014). Another study reported obtaining higher yield of bioactive compounds from onion solid wastes using ethanol and water mixture (60:40 v/v) as an extracting solvent (Khiari et al., 2008). These extraction protocols target isolating highest yields of bioactive compounds while reducing environmental pollution associated with the use of extraction solvents (Câmara et al., 2022; Khataei et al., 2022).

#### 3.1.2 Enzymatic extraction

Enzymatic emerging extraction method uses enzyme during the extraction process. The enzymes act on cell walls resulting in the release of bioactive compounds. Studies have reported that the use of enzymes such as cellulases, proteases and pectinases in the extraction processes accelerated the extraction process leading to excellent yield of bioactive compounds such as phenolic acids and flavonoids (Hung et al., 2020). The enzymatic extraction method is capable of functioning at mild conditions resulting in preservation of the heat-sensitive bioactive compounds (Ben-Othman et al., 2020). Studies have confirmed that this protocol has the capacity to increase the extraction of the antioxidant components (Ghandahari Yazdi et al., 2019). This is supported by the study aimed at obtaining the phenolic compounds from citrus peel through the use of pectinase enzyme. The results showed that the yield was higher compared with the traditional extraction protocols (Hung et al., 2020). However, this protocol is associated with shortfalls such as high cost of the enzymes and setting enzymes optimum performance conditions thereby restricting its industrial applications (Pereira et al., 2023).

#### 3.1.3 Ultrasound-assisted extraction

Ultrasound-assisted extraction (UAE) depends on ultrasound of waves to generate foams in the solvent that instantly induces shock waves leading to destruction of plant cells and change mass transfer (de Aguiar et al., 2024). This approach has proved to be more effective and reliable especially, on extraction period and giving excellent yield (Zia et al., 2023). Various studies have registered success at extracting different bioactive compounds such as polyphenols, flavonoids and carotenoids from various AFW by adopting the UAE technique. A study reported that bioactive compounds extracted from *Moringa oleifera* leaves using UAE demonstrated excellent antioxidant properties compared with bioactive compounds extracted using traditional extraction protocols (Dadi et al., 2019). A separate study indicated that the use of ultrasound during extraction process enhance the release of extractable chemical substances, thereby increasing the extraction effectiveness (Jiménez-moreno et al., 2019).

The percentage yield in the UAE protocol depend on working parameters such as extraction time, temperature and amplitude. These working parameters are recognized to be significant in determining the effectiveness of the extraction process (Bola et al., 2022; Weremfo et al., 2023). Researchers have indicated that changing the operating parameters leads to significant increase in yield. It has been reported that the extraction of the phenolic compounds increases with an adjustment in ultrasound. However, excessive exposure causes degradation of delicate compounds (Sirichan et al., 2022). Still more, there is a need to select appropriate solvents because solvents determine the solubility of the desired compounds as well as influences the overall extraction process (de Aguiar et al., 2024).

#### 3.1.4 Supercritical liquid extraction

Supercritical liquid extraction (SFE) uses supercritical fluids such as carbon dioxide at a high temperature and pressure (Cádiz-Gurrea et al., 2019). Carbon dioxide has unique properties, which include low viscosity, high diffusion rate and possesses solvent-related characteristics. This protocol has demonstrated the ability to achieve high extraction efficiencies with little degradation of delicate bioactive compounds compared with the traditional solvent extraction methods. Phytochemicals, such as phenolic compounds and flavonoids have been effectively obtained from complex agri-related materials (Anticona et al., 2020; Cerón-Martínez et al., 2023). Several research studies have reported that SFE method has been used to obtain multiple bioactive compounds from different plant residues. Furthermore, studies indicate that optimization of extraction parameters assists in increasing the extraction yield and the activity of targeted bioactive compounds. This technique is much more ecologically friendly since it does not involve the use of toxic extraction solvents (Hrelia et al., 2023).

#### 3.1.5 Solid-state fermentation

The solid-state fermentation (SSF) extraction approach involves degradation of lignocellulose biomass with less content of moisture. These operating conditions favors the growth of bacteria and fungi that are capable of producing enzymes (Ding et al., 2019). The enzymes produced have the ability of turning composite compounds into beneficial bioactive compounds such as phenolic acids and antioxidant peptides (Nugraha et al., 2024). This extraction technique can be adapted to different categories of AFW, which in turn, can increase sustainable waste management through valorization (Ezeorba et al., 2024; Yadav et al., 2024). Other research studies have indicated that employing the SSF extraction protocol on AFW can enhance the extraction of vital chemical substances, for instance, chlorogenic acids and antioxidants, suggesting that this protocol increases both the yield and concentration of the bioactive compounds (Frosi et al., 2021; Nugraha et al., 2024). SSF studies done on green peas peel has exposed excellent antioxidants connected with the increased concentrations due to the occurrence of bioactive metabolites, revealing the effectiveness of SSF protocol in obtaining essential compounds (Goodarzi Boroojeni et al., 2017). This protocol is associated with lower operation costs, the capacity to process complex AFW and high product efficiency (Oluremi and Okhonlaye, 2020).

#### 3.1.6 Pressurized liquid extraction (PLE)

PLE is an emerging extraction technique which uses high temperature and pressure to enhance solubility and extraction effectiveness of desired bioactive compounds from different sources of AFW (Barp et al., 2023). This extraction method is also known as accelerated solvent extraction. This protocol has several advantages, such as high extraction yields, shorter extraction duration and prevention of compounds from reactive oxygen species and light exposure (Raut et al., 2015). Studies indicate that this protocol has shown to be effective in the extraction of bioactive compounds such as fatty acids, curcuminoids, lipids, and anthocyanins. Other studies have explored the use of this protocol in the extraction of food contaminants, such as processing and environmental contaminants, veterinary pharmaceutical residues, mycotoxins and pesticides (Barp et al., 2023; Osorio-Tobón and Meireles, 2013; Raut et al., 2015).

#### 3.1.7 Solid-phase microextraction (SPME)

The SPME technique is a rapid protocol that depends on solvent to extract the desired bioactive compounds from AFW. This protocol depends on coated fiber to acquire the analyte from either solid or liquid medium (Jalili et al., 2020). The method has demonstrated to be more efficient and facilitate the extraction of both volatile and semi-volatile compounds. It has shown to possess several benefits, such as cheap, simple and compatible with several analytical instruments compared with traditional extraction protocols (Bagheri et al., 2014; Mottaleb et al., 2014). Additionally, this method has demonstrated to be convenient and reliable for obtaining and quantifying bioactive compounds even at low concentrations. It has been used in several fields such as, environmental science, forensics, applied chemistry and pharmaceuticals. The fusion of this technic with spectroscopic and chromatographic technique has further increased its analytical capacities, rendering it suitable for various settings (Mottaleb et al., 2014).

#### 3.1.8 Purse electric field (PEF) extraction

This is another modern extraction protocol which depends on electrical voltage to break down the cell membrane, thereby releasing the intracellular compounds. PEF extraction protocol is commonly used in the extraction of polyphenols and juice from several sources of AFW (Pappas et al., 2022). Studies indicate that adjusting pulse time and electric field intensity produces high recovery rates of bioactive compounds (Ngadi and Bazhal, 2006). This protocol has demonstrated its effectiveness by increasing the yields of bioactive compounds from various AFW while reducing extraction time (Pappas et al., 2022). Literature reports that the PEF extraction method achieved higher yields compared with traditional methodologies, with 58% and 92% for total polyphenols and specific compounds, respectively (Lin et al., 2005; Yusoff et al., 2017). This extraction technique involves the use of chemical kinetics in order to obtain the preferred chemical compounds. However, it is important to regulate high temperature during the treatment process since enormous heat can compromise extraction efficiency and induce degradation of bioactive compounds (Lin et al., 2005).

The methods discussed thus far are reported to be used more often for the extraction of bioactive compounds from AFW because they have demonstrated efficiency and reliability. However, every method has advantages and disadvantages, as indicated in Table 1, hence studies suggest blending extraction techniques in order to enhance the extraction process. For example, researchers have shown that combining enzymatic and ultrasound-assisted extraction approaches, resulted in excellent extraction yields while decreasing the setbacks associated with each extraction protocol (Buvaneshwaran et al., 2023; Cheikh et al., 2023; Panzella et al., 2020).

The selection criteria of green extraction solvents for attaining bioactive compounds from various AFW comprise several approaches, such as environmental effects, toxicity, cost-effectiveness, biodegradability and extraction efficiency (Patrice Didion et al., 2023; Wu et al., 2022). Efforts by researchers to improve the performance of green solvents have resulted in various developments and innovations by incorporating several operating parameters. Several classes of green solvents have risen recently as viable options, namely bio-based solvents, ionic liquids, supercritical carbon dioxide and deep eutectic solvents (DESs) (Hashemi et al., 2022; Patrice Didion et al., 2023). DESs are quite preferred and favoured due to their easiness in preparation, affordability and greater tenability as opposed to ionic solids (Zainal-Abidin et al., 2019). Researchers have further developed computerised tools such as Hansen solubility parameters and COSMO-SAC modelling devices to enhance extraction processes (Wu et al., 2022). The green solvents are much favoured and used with emerging extraction strategies such as pressurised solvent extraction, microwave-assisted extraction and ultrasound-assisted extraction to increase success while preserving the environmental sustainability (Hashemi et al., 2022; Patrice Didion et al., 2023).

The published reports indicate that green solvents demonstrate best extraction efficiencies as opposed to traditional based organic solvents for attaining bioactive compounds from AFW (Patrice Didion et al., 2023). DES has shown to be remarkable with highest recovery yields ranging 93–99 percent of bioactive compounds than water and traditional organic reagents (Ma et al., 2025). Green solvents offer several benefits including mild and non-destructive properties to delicate compounds during extraction and do not pose threats to ecosystem when discharged in environment (Nakhle et al., 2021). The development and emerging of these technologies techniques provides solutions towards resolving health and environmental concerns related with large quantities of organic solvents. These emerging techniques are promising and reliable compared to traditional techniques (Hashemi et al., 2022).

Traditional and emerging protocols require further exploration in order to resolve some setbacks. Traditional protocols depend on toxic chemical reagents and high energy usage, resulting in pollution and emission of greenhouse gases (Barba et al., 2015; Xi et al., 2021). Low selectivity resulting in difficulty in extracting specific compounds from complex plant matrices and waste disposal challenges, especially toxic byproducts. Traditional strategies are also associated with low recovery rates, especially in acid and heat treatment (Blicharski and Oniszczuk, 2017; Frosi et al., 2021). On the other hand, emerging traditional protocols face issues associated with being quite expensive and market competition, requiring industrial validation and resolving health and safety concerns since the environmental and health effects of novel reagents are unknown (Shahid et al., 2016; Verep et al., 2023).

It is significant that researchers should further explore the possibility of recycling solvents to reduce waste discharge during the extraction process. The blending of traditional and emerging extraction strategies, for instance, acid treatment seconded by bioleaching to boost extraction efficiency and selectivity. Further, there is also a need to employ machine learning in designing experimental extraction parameters to achieve the high recovery yields.

### 3.2 Isolation and purification of bioactive compounds

The purification process of the bioactive compounds obtained from plant sources is carried out for their therapeutic potential and promotes their efficacy for various applications. Studies have reported that bioactive compounds are commonly purified by *solid-liquid* phase extraction, *liquid-liquid* phase extraction and gel purification chromatography. These methods are recognized due to their significant roles in the purification and characterization of the bioactive compounds. In addition, crystallisation and distillation are also recognized as reliable separation and isolation techniques for chemical substances based on their physical properties (Ventura et al., 2017). The selection of the purification method depends on the required purity, yield and activity retention of the targeted bioactive compounds (Kumar et al., 2023; More et al., 2022).

#### 3.2.1 Solid-liquid phase

This technique employs the polar and non-polar solvents to break complex matrix and facilitate the release of bioactive compounds. Research studies have indicated that solvents such as methanol, ethanol and acetone has shown promising results by increasing the extraction yields of phenolic compounds and flavonoids bioactive compounds (Mathew et al., 2023). The process of purification can be optimized through consideration of operating parameters such as, temperature, solvent type and extraction period (Sridhar et al., 2022; Torres et al., 2016). Other studies have recommended that solubility of desired bioactive compounds from the plant matrix sources depends on the solvent which demonstrates the efficiency they can be extracted. Further, other researchers have suggested that performing pre-extraction evaluation studies aiming at attaining high extraction yields and successful purification techniques (Daryani et al., 2022).

#### 3.2.2 Liquid-liquid phase

This purification is less traditional compared with the solid-liquid phase purification approach. Liquid-liquid phase has been demonstrated to be useful in the fractionation of solid plant materials that often involve adsorption methods on solid stationary phases (Staicu et al., 2023). This method is reported to have been used for the removal of undesired plant pigments and chlorophyll, thereby purifying the preferred bioactive compounds (Staicu et al., 2023). The mineral elements adsorbents or chromatographic materials used permit the retaining of selected materials and elution of the desired plant compounds, thereby separating the high-grade compounds that can show excellent bioactivity. Further, studies indicate that solid-solid phase yields highly concentrated fractions of bioactive compounds (Alaekwe et al., 2023).

#### 3.2.3 Crystallization

Crystallization protocol of bioactive compound purification is considered a significant protocol for obtaining high-purity products. It operates on the idea of different solubility’s of compounds in the mixture, providing an opportunity for crystallization of the preferred product. However, studies have indicated that the presence of chemical contaminants in the sample can greatly affect the yield and the purity of the crystalline product (Balint, 2001; Luo and Shen, 1987). Researchers have resolved this challenge through the development of hybrid techniques by integrating chromatography and crystallization techniques. This protocol operates based on the capacities of both techniques, resulting in more effective separation and purification of desired bioactive chemical substances from complex mixtures (Balint, 2001). For instance, the isolation of artemisinin from *Artemisia annua* was possible through the incorporation of HSCCC with crystallization, resulting in the yield of bioactive compounds with high purity and synergistic properties (Qu et al., 2010).

#### 3.2.4 Distillation

Furthermore, distillation is another technique that is also used in the purification of bioactive compounds. However, regardless that this protocol is less generally associated with purification of bioactive compounds, it can, though, be used, more especially for obtaining volatile compounds. It operates based on differences in boiling points of desired bioactive compounds in the mixtures. Studies have reported that essential oils and volatile organic bioactive compounds were obtained successfully from AFW while maintaining the desired benefits (Gontarek-Castro and Lieder, 2021; Mahrous and Farag, 2022). Further, this method favours bioactive compounds that are thermally stable and can be evaporated without disintegration. Therefore, this implies that distillation protocol is commonly used in combination with other purification techniques to obtain bioactive compounds with high purity and yield (Madureira et al., 2021).

#### 3.2.5 Chromatography

Chromatography is one of the most used methods for purifying bioactive substances. Chromatography can be categorized based on procedures, such as high-performance liquid chromatography (HPLC), countercurrent chromatography (CCC) and affinity chromatography, developed based on specific types of molecules and matrices. For example, high-speed countercurrent chromatography (HSCCC) is popular because it can separate bioactive compounds without them sticking permanently to the materials used in regular solid-phase chromatography (Han et al., 2012). This method is specifically significant for isolating bioactive substances from complicated mixtures, such as plant extracts and the components may affect the separation process (Tian et al., 2024). The HSCCC permits high recovery rates and is suitable for large-scale operations, recommended for researchers that specialize in natural plants (He et al., 2018).

The type of chromatography technology used is frequently determined by the nature of bioactive compounds targeted. Recent research studies indicate that phenolic compounds have gained attention due to their antioxidant properties. These compounds are reported to be isolated and purified through the use of various chromatographic procedures, for instance, supercritical fluid chromatography and medium-pressure liquid chromatography (Susanti et al., 2024). These methods have demonstrated to enhance purity and biological activities by promoting the phytochemical profile during the extraction process (Cano-Gómez et al., 2024). Additionally, studies have shown that integrating several bioactivity-guided purification protocols can also promote enhancing the isolation process, thereby permitting researchers to focus on molecules with specific health benefits (Mathew et al., 2023).

##### 3.2.5.1 Gel filtration chromatography

This technique is also termed as size exclusion technique has working principle based on differences in molecular size (Fagain et al., 2016). The technique uses a packed column containing porous beads that inhibit large molecules exclusion from the pores, and they are obtained first, whereas smaller molecules penetrate the pores and are eluted later (Masoodi et al., 2021). Studies report that this protocol has shown increased effectiveness and reliability, especially towards in handling complex matrices containing proteins and peptides (Fashakin et al., 2023). This method has demonstrated its capacity in purifying hydrolysate protein molecules while sustaining its functional properties, revealing its uniqueness (Kasiwut et al., 2015). Additionally, the other similar studies have revealed that smaller molecular weight compounds, such as antioxidants, are obtained from protein hydrolysate while maintaining bioactivity characteristics (Sasidharan et al., 2011). Other studies have reported that incorporation of this protocol with ion-exchange chromatography has revealed to be significance in attaining well-purified fractions from complicated plant matrices (Zou et al., 2014).

##### 3.2.5.2 Thin layer chromatography

Research studies report that various bioactive compounds obtained from several plant sources are indicated to be isolated and purified with thin-layer and column chromatography (Hameed et al., 2023). Column chromatography and thin-layer chromatography (TLC) remain the most reliable and convenient owing to their easiness, affordability and existence of various stationary phases. The most commonly used agents in phytochemical separation in the stationary phase are alumina, silica, cellulose and polyamide (Preethi et al., 2017). Studies reported that the occurrence of complex matrices in phytochemicals has made separation a challenging task. TLC has been used for decades to determine compound fractions using column chromatography. Studies have reported that bioactive compounds have been separated successfully with the help of silica-gel column chromatography and TLC together with other analytical tools (Sherma, 2013; Sherma, 2014).

The isolation and purification processes of bioactive compounds have resulted into significant discoveries and associated setbacks. It is recommended that future research should focus on improving shortfalls associated with these isolation and purification techniques. The solvents used in liquid-liquid and solid-liquid phases for purification processes are toxic, expensive and threats to the environment and ecosystems (Wang et al., 2023). Future studies should focus on developing solvents with advanced properties that can be used for purification without causing much damage to the environment (Pai et al., 2022). There is a need to focus on improving distillation and crystallisation techniques and developing solvent-free methods for isolation and purification driven by machines. The chromatography technique is generally affected by several issues; for instance, reliance on toxic solvents, expense, batch inconsistencies and prolonged processing durations affect efficiency (Custodio-Mendoza et al., 2024; Hurkul et al., 2024). Furthermore, future studies should focus on employing advanced hybrid chromatographic systems in order to increase selectivity and reduce costs. The environmental issues associated with solvent usage can be resolved if studies explore developing less solvent consumption techniques while maintaining bioactivity of the extracts.

### 3.3 Structural characterization of bioactive compounds

Bioactive compounds obtained from natural sources and food products are commonly characterized through the use of several analytical techniques. These methods are categorized into traditional and spectroscopic techniques. Traditional protocols are photometric techniques and chromatographic protocols such as TLC, HPTLC, HPLC and GC (Debnath et al., 2024; Horszwald and Andlauer, 2011). On the other hand, spectroscopic modern characterisation techniques encompasses methodologies such as FTIR, NMR, UV-visible spectroscopy and Mass spectrometry that reveals structural information (Debnath et al., 2024; Saxena, 2023). Studies have recognized the use of LC-MS and GC-MS for identification and determination of bioactive compounds (Jeszka-Skowron et al., 2015). The use of microplate in characterisation of bioactive compounds offers several benefits such as reagent savings and time efficiency as opposed to conventional cuvette methods. These analytical techniques are used to identify several bioactive compounds such as flavonoids, polyphenols, caffeine and chlorogenic acids in various matrices such as berries and coffee (Horszwald and Andlauer, 2011; Jeszka-Skowron et al., 2015). These methods operate on different principle that depends on interaction of electromagnetic radiation or mass-to-charge ratios with matter to indicate molecular structures.

#### 3.3.1 Infrared spectroscopy

This characterization technique of bioactive compounds depends on molecular vibrations to absorb infrared radiation. The bioactive molecules exposed to infrared radiation, acquires specific wavelengths that links to the vibrational modes within the molecule (Manzoli, 2019). The emitted spectrum acts as a molecular fingerprint region often used to detect and identify the functional groups and molecular conformations. This technique is suitable for identifying polar covalent bonds with higher dipole moments and so enhance vibrational transitions (Csizmar et al., 2012). Fourier-transform infrared (FTIR) spectroscopy enhances the ability by transforming time-domain information spectra, which promotes resolution and sensitivity (Csizmar et al., 2012; Manzoli, 2019). This method is generally used in molecular characterisation due to its uniqueness in revealing and providing information about functional groups, molecular interactions and conformations transformations, all of which are relevance for appreciating and understanding biological activity (Saurabh and Mukamel, 2016). Studies have shown that bioactive compounds obtained from fruit and vegetable wastes (apple peels, carrot peels, beetroot peels, and potato peels) were successfully characterised by FTIR. The functional groups, such as C-O, O-H, C=O, and N-H, were detected, which were associated with starchy carbohydrates, organic acids, or proteins (Filip et al., 2024).

#### 3.3.2 UV-visible spectroscopy

**FIGURE 3:**
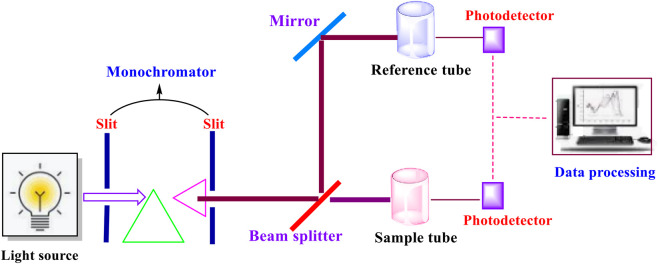
Scheme of UV-Spectroscopy.

The UV-visible spectroscopy analytical technique operates on the principle of electronic transitions within molecules (Figure 3). Whenever samples of bioactive compound molecules absorb ultraviolet or visible light, induced electrons get excited, thereby jumping from their lower energy state to a greater energy state (Begum et al., 2018). Through this transitioning process, the electrons emit radiations in the form of wavelengths of light absorbed that are associated with molecular structure, thereby resulting in the identification of conjugated systems and chromophores (Karmakar et al., 2021). This technique is preferred and favoured for studying and detecting molecules possessing π-electron bonds, for instance, polyphenolic and flavonoids, among others. These are also found in other bioactive chemical compounds, as indicated by other researchers (Begum et al., 2018). The produced absorbance spectrum can be quantitatively studied through applications of Beer-Lambert’s equation, resulting in the generation of data associated with the concentration of the analyte in the solution. This suggests that the UV-visible spectroscopy protocol is relevant for detecting and analyzing the purity and concentration of the obtained bioactive substances (Begum et al., 2018; Sirajuddin et al., 2013). Further, this protocol can also be extended in the monitoring of chemical interactions amongst the molecules (Sirajuddin et al., 2013). Another study has reported that phenolic compounds obtained from AFW were successfully profiled by UV-visible spectroscopy (Mir-Cerdà et al., 2023).

#### 3.3.3 Nuclear magnetic resonance spectroscopy

Nuclear magnetic resonance (NMR) spectroscopy, a non-destructive, reliable and convenient technique, employs magnetic characteristics for certain nuclei. The moment the sample is placed in a high magnetic field, the nuclei vibrate at specific frequencies, which are determined by their chemical surroundings. The vibration is recognized and turned into a spectrum that comprises data about the compound’s molecular structure and dynamics as well as interactions (Kokab et al., 2021; McDermott et al., 2002). NMR is special analytical tool for structure elucidation due to its unique precision information regarding atom connectivity and stereochemical configurations (Du et al., 2021; Robien, 2019). The NMR technique has multiple uses across several fields of investigations of bioactive compounds since it can indicate complicated structures, especially those of natural products and medicine (Robien, 2019). Additionally, studies indicate that recent advances in NMR techniques, such as two-dimensional NMR, have been demonstrated to enhance the flexibility of biomolecules and their interactions (McDermott et al., 2002). A study recently published the use of NMR analytical technique in the characterisation of bioactive compounds (d-limonene, hesperidin, and valencene) extracted from orange juice waste using the MAE technique. The NMR results indicated the attainment of high-purity hesperidin compound with 87.66% (Penteado et al., 2025). The study demonstrated the significance of the NMR analytical tools in the characterisation of bioactive compounds from AFW.

#### 3.3.4 Mass spectroscopy

**FIGURE 4:**
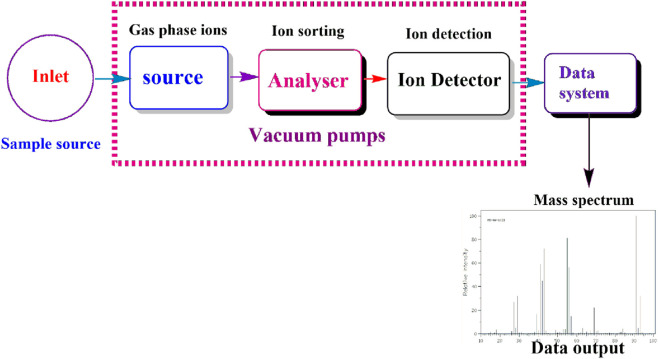
Scheme for mass spectroscopy.

Mass spectroscopy (MS) is another effective and reliable analytical technique used for evaluating the mass-to-charge ratio of ionized particles during the characterization process of bioactive compounds (Thakur, 2020). The significant principle is based on ionization of chemical species to generate charged molecules or fragments, which are subsequently separated in a vacuum based on their mass-to-charge ratios (Brown et al., 2020; Picó, 2020). The resulting mass possesses data concerning the analyte’s molecular weight and structural characteristics, as summarized in (Figure 4), (Géhin and Holman, 2021). MS can also be integrated with chromatographic protocols such as LC-MS aiming at promoting separation and identification capacities for bioactive chemical substances (Picó, 2020). The combination of these protocols has demonstrated to reduce the challenges associated with the study of complicated mixtures, especially those observed in biological samples, making it easier to detect metabolites and other bioactive compounds even at reduced concentrations (Soleiman-Beigi and Ghiasbeigi, 2019). The study done by Abbattista et al. (2021) demonstrated that MS is a useful analytical instrument in the characterisation and identification of secoiridoids and phenolic compounds from olive leaves and pomace wastes. This technique provides an opportunity in targeted and untargeted compounds offering comprehensive characterisation for complex waste extracts.

### 3.4 Bioactive compounds from agri-food wastes

**FIGURE 5:**
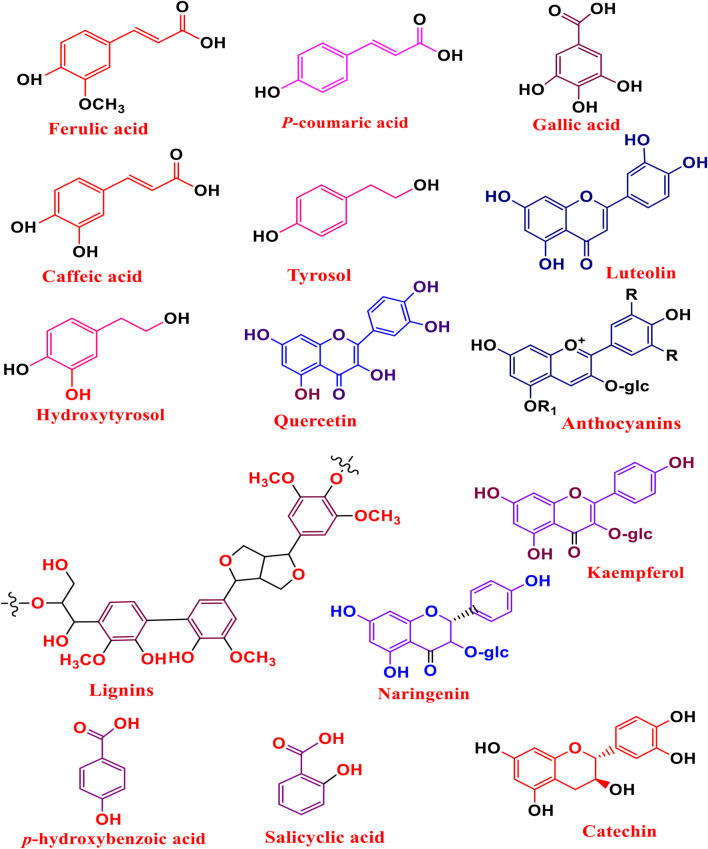
Examples of phenolic compounds extracted from AFW.

There are several phenolic compounds that have been successfully extracted and purified from various agri-food wastes. Researchers have obtained these essential bioactive compounds from fruit peels, vegetable leaves, cereal bran and tea waste. Studies have demonstrated that AFW, such as orange and lemon peels, rice husks, onion peels, carrot peels, apple peels, tomato peels, potato peels, olive waste, grape waste, wine products, have been shown to possess various phenolic compounds presented in (Figure 5). These AFW residues are also reported to contain high levels of polyphenols, flavonoids and vitamins which are associated with several health-promoting benefits, such as anti-inflammatory and anti-diabetic properties. These phenolic compounds are obtained through the use of various extraction protocols such as UAE, MAE and DES among others. Additionally, phenolic bioactive compounds are recognized for their unique various industrial applications including food, pharmaceuticals and cosmetics (Ben-Othman et al., 2020; Ligianne and Ceccato-Antonini, 2020; Sorrenti et al., 2023; Yadav et al., 2024).

Studies have indicated that these phenolic compounds can be obtained from AFW through the use of emerging extraction protocols. These methods are much preferred and favored due to their uniqueness such as high extraction yields, short extraction periods and less consumption volume of solvents compared with traditional protocols. The most widely used emerging techniques for obtaining phenolic compounds from various sources of AFW sources are UAE, SFE, DES, MAE and MGH (Bhadange et al., 2024; Panzella et al., 2020). Studies have indicated that these methods are convenient for higher concentration yields of polyphenols with high antioxidant properties obtained from various AFW. The higher yields, concentrations and purity of phenolic compounds obtained from AFW depend on operating conditions. Researchers have recommended that to attain higher yields and desired results, there’s a need to select proper extraction conditions, extraction methods and resolve shortfalls associated with these characterisation techniques (Mir-Cerdà et al., 2023; Panzella et al., 2020; Zhou et al., 2021).

The analytical instruments used in the characterisation of bioactive compounds obtained from agri-food wastes face some gaps that require further exploration for improved results and major breakthroughs in scientific disciplines. The MS spectroscopy tool is expensive, and there is complexity in data analysis and matrix effects that hinder detection, especially in complex plant matrices (Susanti et al., 2024). Further, variation in standardisation methodologies remains unclear, resulting in variation in studies across (Abedelmaksoud et al., 2025). Studies should focus on integrating complementary instruments such as MS and NMR techniques for structural elucidation, explore the possibility of machine learning in data interpretation and develop high-sensitivity and reliable systems for food analysis. On the other hand, UV-visible spectroscopy is recognised for effective screening of chromophoric compounds such as carotenoids and phenolic compounds (Kharbach et al., 2023). However, the instrument is associated with overlapping of absorption spectra and is prone to interference from other food matrix components, which hinders correct quantification of food samples. Studies also indicate that temperature compromises the reproducibility of results (Saxena, 2023). Future studies should focus on integrating chemometric tools to enhance resolution and also develop portable tools for on-site testing in order to increase bioactive compound profiling.

Finally, FTIR is well known for detecting various functional groups existing in different foodstuffs. However, the use of this instrument is known to be associated with setbacks such as low selectivity for low concentration analyses, overlapping of spectra, especially in heterogeneous mixtures, and the presence of water has also been demonstrated to cause interference (Orehek et al., 2021). Additionally, another setback is non-compatibility in comprehensive structural sequencing (Falsafi et al., 2025). It is suggested that future studies should adopt machine learning in data processing and spectra deconvolution. Lastly, integrating several analysis protocols through multimodal platforms such as FTIR with UV-Vis spectroscopy or MS.

### 3.5 Challenges associated with isolation, purification and characterization from AFW

Scalability of bioactive compounds derived from AFW for various industrial application processes faces some challenges such as extraction, purification and characterisation processes, as reported in the literature. On top of that, their usage is associated with setbacks, such as the non-existence of standardisation and extraction protocols, regulatory barriers and consumer acceptance (Chauhan & Kumar, 2023). There are several bodies which were established mainly for monitoring and issuing acceptance of food substances on markets to safeguard the wellbeing of the consumers. These bodies are the European Food Safety Authority (EFSA), the United States Food and Drug Administration (U.S. FDA) and the Food Safety and Standards Authority of India (FSSAI), among others. However, EFSA is the primary body responsible for permitting and approving the use of bioactive compounds, while other markets have established their own national regulatory standards. Furthermore, safeguarding the chemical stability of acquired compounds and dealing with technological challenges still remain the major challenge (Simões et al., 2021).

The major challenge is about the difficulty of the plant matrices and the existence of a variety of phytochemicals that require proper extraction protocols to effectively obtain preferred bioactive compounds (Alamgir, 2018). The proper selection of the extraction technique significantly determines the purity and yield of the bioactive compounds, with careful solvent selection taken into consideration. For instance, one study reported successfully extracting 4-ethylheptyl benzoate from Rumex nervosus roots through the use of petroleum ether and methanol solvents. However, the use of the single solvent, petroleum ether, in the extraction of the same bioactive compound was unsuccessful (Nigussie, 2020). Studies indicate that the process of extracting bioactive compounds is affected by their interaction in the cell structures, particularly if it involves the phenolic chemicals which are found in algae organisms; their extraction process needs cell destruction for an efficient and successful extraction process (Besednova et al., 2020).

Additionally, the purification process generally follows several chromatographic stages, such as column chromatography and preparative TLC, which can be labour intensive and may result in the damage of bioactive compounds or even contamination if not accurately optimised (Nigussie, 2020; Wali et al., 2024). Attaining high-purity bioactive compounds is significant for successive characterisation and biological activity studies. However, this remains the primary setback due to the occurrence of structurally similar compounds and impurities that cause interference. The characterisation tools, especially spectroscopic methods such as NMR, UV-VIS and FT-IR, are necessary but need advanced instrumentation and expertise, which can be a challenging factor, especially in resource-limited countries (Alamgir, 2018; Nigussie, 2020).

Furthermore, another significant setback is the stability of bioactive compounds during the extraction and purification processes. Studies indicate that some phytochemicals are delicate to environmental conditions such as light, pH and temperature, which can cause degradation or structural damage (Alamgir, 2018). Still more, the presence of different phytochemicals in AFW sources makes it hard to develop standardised protocols for extraction and purification of bioactive compounds. The variation in AFW requires adaptable and robust techniques to ensure reliable isolation of bioactive compounds (Khanal et al., 2023).

Lastly, the characterisation stage requires resolving the biological activities of the obtained compounds, which uses bioassays to confirm efficiency. The bioassays used at times can be complex and need specific operating conditions to correctly measure the specific activity, such as antimicrobial and antitumor effects, adding another layer of challenge in the overall process (Alamgir, 2018; Ezeorba et al., 2024). In general, the extraction, isolation, purification and characterisation of bioactive compounds from AFW encounter setbacks such as protocol complexities, resource requirements and compound stability issues as well as source variability, thereby encouraging continuous studies to advance more reliable, efficient and standardised techniques (Alamgir, 2018; Besednova et al., 2020; Nigussie, 2020).

### 3.6 Applications of bioactive compounds

The bioactive compounds obtained from AFW are currently being explored in various industrial sectors such as pharmaceuticals, cosmetics and functional food additives in food processing and packaging for various uses, as indicated in (Table 2). The use of AFW has positive outcome such as decreasing the environmental issues associated with AFW disposal, utilisation of phytochemical compounds from plants and offering employment especially to the youth residing in remote areas where inhabitants depend on agricultural activities for livelihood. The extracted bioactive compounds can boost and enhance economic growth in the pharmaceutical, food and cosmetic industries (dos Santos et al., 2018; Manousaki et al., 2016).

**TABLE 2:** Source of bioactive compounds and their industrial applications.

No	AFW	Bioactive obtained	Industrial applications of bioactive compounds	References
1.	Apple pomace	Polyphenol, Fiber	Functional food additives	Grispoldi et al. (2022)
2.	Apricot pulp waste	Antioxidant polyphenols Carotenoids	Health and wellness Nutraceuticals Dietary supplements	Chatzimitakos et al., 2023
3.	Agri-byproducts	Vitamins Carotenoids	Food supplements Cosmetics	Zhou et al. (2021)
4.	Fruit and vegetable Waste	Carotenoids Vitamins Polyphenols	Pharmaceuticals Cosmetics Food enrichment	Sagar et al. (2018)
5.	Cereal bran	Dietary fibers Polyphenols	Functional food ingredients Animal feed	Tufail et al. (2022),Plakantonaki et al. (2023)
6.	Food-Processing Byproducts	Phenolics Essential oils Antioxidants	Nutritional supplement Food preservation	Castrica et al. (2019),Malenica et al., 2023
7.	Chestnut burrs	Antioxidants Ellagic acid	Antimicrobial supplements Antimicrobial supplements	Trezza et al. (2024)
8.	Mango and Guava Byproducts	Antioxidants Dietary fibers	Pharmaceuticals Functional ingredients Nutraceuticals	Cerón-Martínez et al. (2023)

#### 3.6.1 Pharmaceutical applications

Recent scientific research has focused on development of drugs using chemical compounds extracted from AFW. It has been reported that phenolic compounds obtained from food related materials are capable of disrupting biological pathways associated with several disorders, rendering them ideal therapeutic substances (Ivanišová et al., 2012; Khan et al., 2020; Mulugeta and Samuel, 2022). For instance, studies indicate that benzimidazole derivatives are associated with biological properties such as antibacterial and anticancer characteristics, suggesting their significance as possible major molecules for the development of new therapeutic drugs (Mulugeta and Samuel, 2022). Still more, the high incidence of non-communicable disease has initiated the interest in bioactive compounds as viable and reliable protective and therapeutic cures, thereby enhancing high investment in pharmaceutical research and development (Khan et al., 2020; Martirosyan and Miller, 2018).

#### 3.6.2 Cosmetics use

Research studies have confirmed that the use of bioactive compounds in cosmetic industries has significant benefits. It has been shown that the ingredients originating from AFW such as antioxidants, natural pigments and anti-inflammatory agents, are presently being added to skincare and cosmetic products to enhance efficiency and appeal to the users (Ghosh et al., 2015; Álvarez-Castillo et al., 2023). Studies have further shown that the astaxanthin chemical compound found in marine plants and used as an ingredient for skin protection and promoting attractive characteristics in cosmetics industry (Álvarez-Castillo et al., 2023). The use of compounds extracted from plant residues in cosmetic industry assist in resolving consumers choices for clean products that are reliable and safe compared with synthetic chemicals (Sharma et al., 2021). Additionally, the use of chemical compounds of plant origin are significantly favoured due to their functional characteristics, which addresses skin issues such as skin aging, inflammation and pigmentation (Ghosh et al., 2015). These compounds also help in boosting interaction of cellular tissues to enhance their efficiency in promoting skin health and beauty thereby offering affordable and safe alternatives in cosmetic interventions (Duque-Soto et al., 2022). Cosmetic industries are actively engaged in exploring bioactive compounds that possess aesthetic benefits while preserving general skin health.

#### 3.6.3 Food additives and supplements

The bioactive compounds derived from various AFW such as fruit peels, leaves, vegetables, cereals and pomace and other plant-related residues have also been explored in food sector due to their health benefits as they possess high content of phytochemicals such as flavonoids, carotenoids and polyphenols (Sagar et al., 2018; Shawky et al., 2025; Zaky et al., 2024). Studies have also confirmed that AFW are incredible sources of bioactive compounds that contain anti-inflammatory, antioxidant and antibacterial characteristics that reduces the risk of chronic infections such as cancer, diabetes and cardiovascular (Ivanišová et al., 2012; Khan et al., 2020; Rojas et al., 2016). Moreover, research has indicated incorporation of phytochemicals ingredient functional foods especially processed foods ensures consumption of clean and safe food products with ideal health benefits (Lankanayaka et al., 2024; Martirosyan and Miller, 2018). Another study has indicated that bioactive compounds extracted from grape byproducts have shown to contain high levels of antioxidant properties making them suitable for promoting food quality and safety (Jara-Palacios, 2019; Rodrigues Machado et al., 2023).

### 3.7 Recommendations for future research

Optimisation of extraction and purification advancements should target the lowering of the energy use, decreasing the usage of harmful solvents and increasing the recovery rates of the desired bioactive compounds, including proteins, dietary fibres, polyphenols and vitamins. In addition, the use of modern extraction techniques should be further revised to increase the yield and scalability while lowering the environmental effects (Yadav et al., 2024).

It is also suggested that the exploration of the use of pretreatment methods such as physical, chemical and biological methods can help to promote high yields of targeted bioactive compounds. The use of physical methods, such as grinding or heating, increases opportunities for accessing substrates (Peguero et al., 2022). Also, the use of chemical reagents such as alkali, facilitates the breakdown of complex polymer chains found in AFW residues. Similarly, the use of microorganisms such as fungi and bacteria to break down resistant complex material substances (Hadj Saadoun et al., 2021). Further studies are required to explore the most effective pretreatment combination for various categories of AFW and the desired bioactive compounds.

Moreover, the use of innovative biological techniques such as solid-state fermentation technique is encouraged. This technique employs the use of microorganisms such as bacteria, yeast and fungi for the production of value added products derived from AFW (Berenguer et al., 2023). These microorganisms grow on solid materials in the absence of flowing water as used for flavour production. Studies should focus on the selection of appropriate healthy bacterial strains and optimising process working conditions for higher yields and low costs (Kotsou et al., 2024).

In addition, it is suggested that the contemporary research should focus on prioritising the identification and isolation of emergent significant compounds such as the plant-derived extracellular vesicles (PDEVs) with possible applications in therapeutics and nutraceuticals. There’s a need to further explore the scalability and isolation of PDEVs biomolecules from AFWs (Latella et al., 2024; Rodrigues et al., 2022).

Finally, future studies should focus on the alignment of the circular economy, emphasising on sustainable waste reduction and usage and building of closed-loop systems that deal with continuous repurposing of AFW into novel products (Musembi et al., 2024).

## 4 Conclusion

In summary, this review study has found that AFW are rich sources of important bioactive compounds, which are ideal for various industrial applications. The bioactive compounds are obtained through the application of extraction, isolation, purification and characterisation methods. These methods have demonstrated great improvement for the isolation of bioactive compounds, thereby offering an opportunity for their exploration in various fields and industries. This review paper indicates that extracted, purified and characterised bioactive compounds such as phenolic compounds, flavonoids, vitamins, dietary fibre and carotenoids provide reliable and convenient solutions towards waste valorisation. The finding of alternative use for AFW does not only resolve environmental issues associated with AFW disposal challenges but also offers new opportunities for the development of value-added food products in various industrial sectors. The industries of interest are those involved in food additives, nutraceuticals, cosmetics, antioxidants or preservatives and drug discovery. The results obtained from this review study encourage and promote the re-utilization of AFW and recognise them as important source of bioactive compounds.
